# Machine Learning-Driven Probability Scoring Enhances Diagnostic Certainty and Reduces Costs in Suspected Periprosthetic Joint Infection

**DOI:** 10.3390/diagnostics16040626

**Published:** 2026-02-20

**Authors:** Jim Parr, Van Thai-Paquette, Amy Worden, James Baker, Paul Edwards, Krista O’Shaughnessey Toler

**Affiliations:** 1 Data Science and Machine Learning, Zimmer Biomet, Swindon SN5 6NX, UK; james.parr@zimmerbiomet.com; 2 Diagnostics Research and Development, Zimmer Biomet, Claymont, DE 19703, USA; van.thai-paquette@zimmerbiomet.com; 3 Health Economics, Zimmer Biomet, Centennial, CO 80015, USA; amy.worden@zimmerbiomet.com; 4 University of Louisville Physicians—Orthopedics, University of Louisville Health, Louisville, KY 40202, USA; james.baker@uoflhealth.org; 5 Bowen Hefley Orthopedics, Little Rock, AR 72207, USA; paul.edwards@bowenhefleyortho.com

**Keywords:** hip, knee, total joint arthroplasty, periprosthetic joint infection, machine learning, diagnosis, biomarkers, synovial fluid

## Abstract

**Background**: Accurate diagnosis of periprosthetic joint infection (PJI) remains challenging, particularly in culture-negative and borderline cases where current practices lead to high diagnostic uncertainty. SynTuition™, a machine-learning-based probability score integrating preoperative biomarkers, was developed to support clinical decision-making. This study compared its diagnostic performance and economic impact with standard physician practice. **Methods**: A total of 12 physicians provided diagnoses of 274 clinical vignettes representing suspected PJI cases. SynTuition probabilities were converted to binary diagnostic classifications using a validated threshold. Diagnostic accuracy, agreement, indecision rates, decision curve analysis, and misdiagnosis-related costs were evaluated. **Results**: SynTuition achieved an overall percent agreement of 96.0% when compared against the expert adjudicated clinical reference, outperforming the pooled physician group at 90.8%. Physicians showed high indecision (38–48%) in inconclusive 2018 ICM cases, whereas SynTuition generated a definitive diagnosis with an 86.7% agreement against expert adjudication. Decision curve analysis demonstrated a higher net benefit for SynTuition across a broad range of thresholds, reducing projected unnecessary revision by up to 5.8%. Economic modeling showed a reduction in misdiagnosis-related costs from $6.9 million to $2.9 million per 1000 suspected PJI cases, yielding estimated savings of $4000 per suspected case. **Conclusions**: SynTuition demonstrated high diagnostic accuracy, lower uncertainty, and significant clinical and economic advantages over routine physician practice, supporting its integration into clinical decision-making for suspected PJI, particularly in diagnostically ambiguous cases.

## 1. Introduction

Periprosthetic joint infection (PJI) is a serious complication of total joint arthroplasty (TJA), affecting approximately 1–2% of primary joint replacements [1] and representing one of the leading causes of revision surgery [2,3]. As the number of TJAs continues to rise, the incidence of revision surgeries due to PJI is expected to increase correspondingly, imposing a growing burden on healthcare systems and negatively affecting patient outcomes [4].

Missed or delayed diagnosis of PJI can result in failed aseptic revisions, multiple subsequent surgeries, prolonged antibiotic use, and extended hospital stays [3]. Conversely, overdiagnosis may lead to unnecessary revision procedures and antibiotic therapies. Accurate diagnosis of PJI is essential to guide appropriate treatment decisions, and early detection helps to optimize patient selection for less invasive management strategies, such as debridement, antimicrobial therapy, and implant retention (DAIR) [5].

Over the past decade, efforts to standardize the diagnosis of PJI have led to the development of several criteria-based systems, including those proposed by the Musculoskeletal Infection Society (MSIS) [6], the International Consensus Meeting (ICM) [7], the European Bone and Joint Infection Society (EBJIS) [8], and the Infectious Diseases Society of America (IDSA) [9]. These systems integrate clinical findings and laboratory biomarkers to classify PJI and have greatly improved standardization in research. However, challenges persist in applying these criteria consistently in clinical practice [10,11]. A particular difficulty arises in inconclusive or borderline cases, often associated with culture-negative infections, where diagnostic ambiguity increases the risk of both overdiagnosis and underdiagnosis [12].

Given the critical importance of timely and accurate PJI diagnosis, and the limitations of current threshold-based criteria, there is growing interest in artificial intelligence (AI) to augment clinical decision-making [13,14]. Multiple modeling families have been explored for PJI diagnosis, including linear models, tree-based models, support vector machines, and neural networks, each offering different tradeoffs in terms of interpretability, calibration, robustness to class imbalance, and performance on heterogenous biomarker inputs [14,15,16,17,18,19]. Prior studies have shown that ML-based classifiers can outperform established criteria such as the 2018 ICM, but many of these models are limited by small, single-center cohorts and lack external validation, reducing their translational potential [15,18]. In contrast, the SynTuition™ Score (hereafter SynTuition) is the only ML model for PJI diagnosis that has been deployed into routine clinical use, implemented at a centralized U.S. clinical laboratory to provide nationwide access to probabilistic PJI assessment. SynTuition was trained and validated on one of the largest synovial biomarker datasets available, incorporating multianalyte synovial fluid markers, and it has demonstrated strong discrimination across a diverse patient population [19].

Unlike conventional definitions that categorize patients using fixed cutoffs, SynTuition generates a continuous, patient-specific probability of PJI along with actionable, validated decision guidelines. This approach enables explicit quantification of diagnostic uncertainty and alignment with clinical priorities, such as ruling out infection prior to single-stage, aseptic revision surgery or ruling in infection before a two-stage revision. Although this high level of discrimination enables confident diagnostic decision-making, this characteristic is an outcome of its ability to leverage high-dimensional biomarker interactions learned from a large, comprehensive dataset rather than a restriction or requirement driven by the model developers. Hence, SynTuition employs the same pattern recognition principles embedded in all existing PJI definitions and yields clearer decision support in a greater proportion of cases without overriding clinician judgement [19].

The primary aim of this study was to evaluate the clinical impact of SynTuition. SynTuition was compared against physician diagnoses of PJI consistent with their routine clinical practice, utilizing the current standard of care (SOC). Two primary outcomes were assessed in this comparison: the reduction in diagnostic uncertainty and the level of agreement with a widely accepted, expert-endorsed framework. Clinical utility was evaluated using decision curve analysis, and the economic impact was assessed using a decision-analytic model.

## 2. Materials and Methods

### 2.1. Study Overview

This retrospective study performed a secondary analysis of a previously conducted survey study to evaluate the clinical impact of SynTuition. The work built on three prior investigations. First, clinical information and biomarker data were collected from a multi-institutional cohort [20]. Second, these data were used to generate 277 clinical vignettes, each assigned a clinical reference diagnosis using the 2013 MSIS criteria through adjudication by a panel of three experts. Survey responses were collected from 12 physicians who reviewed the vignettes [10]. Third, the SynTuition model was developed and validated in a large, independent cohort [19].

**Figure 1:**
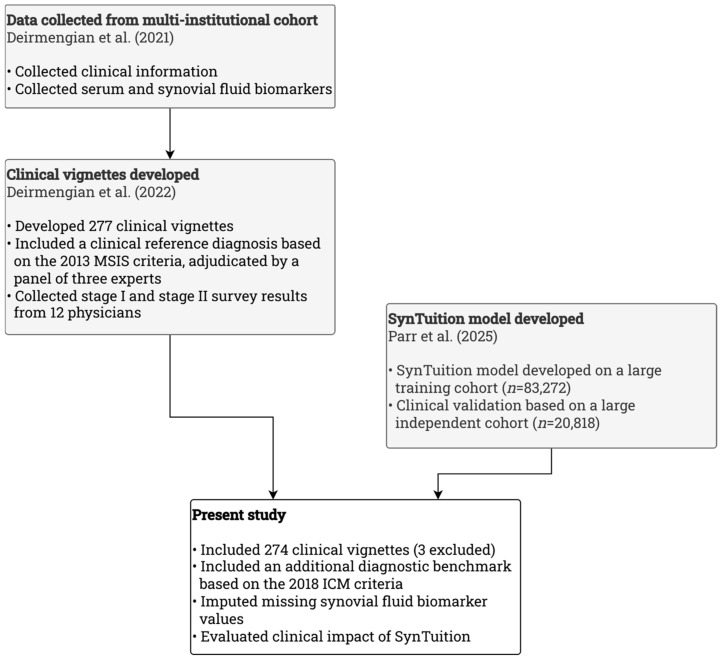
Flow diagram illustrating how the present study used previously derived clinical vignettes and data from earlier studies. The grey boxes highlight earlier investigations by Deirmengian et al. [10,20] and Parr et al. [19].

The present study included 274 of the previously developed vignettes and introduced an additional diagnosis benchmark based on the 2018 ICM criteria for PJI. Missing synovial fluid (SF) biomarker values were imputed, and the SynTuition model was applied to assess its diagnostic accuracy and economic performance. Figure 1 illustrates how the present study was derived directly from these earlier investigations.

### 2.2. Clinical Vignettes

In the prior work by Deirmengian et al., 277 clinical vignettes were created using data collected from a multi-institutional cohort, audited by a contracted research organization [10,20]. Each vignette included clinical information such as the patient age, sex, joint (hip or knee), laterality, concurrent antibiotic use, and comorbidities. The availability of preoperative serum and SF biomarkers varied across each vignette, depending on the diagnostic work-up performed. This reflected the typical biomarkers obtained as part of routine clinical evaluation and included the erythrocyte sedimentation rate (ESR), C-reactive protein (CRP), synovial fluid white blood cell count (SF-WBC), synovial fluid polymorphonuclear percentage (SF-PMN%), and synovial fluid culture results. Each vignette also included postoperative results, including tissue culture and histology, but these were not available in the physician-facing vignettes to enable the assessment of real, clinical preoperative decision-making. Synovial fluid red blood cells (SF-RBCs) and alpha-defensin (AD) results were also available but not included in the physician-facing vignettes, as these biomarkers are not part of the 2013 MSIS definition of PJI used in the original survey study [10].

Deirmengian et al. assigned a clinical reference diagnosis based on the 2013 MSIS definition of PJI [10]. This diagnosis was adjudicated by a panel of three experts until consensus on a definitive classification was reached for each patient. Throughout this manuscript, this adjudicated 2013 MSIS-based classification is referred to as the clinical diagnosis and is used as the primary ground truth label, as it represents the best available clinical truth in the absence of a gold standard.

To provide an updated benchmark, each vignette was additionally classified according to the 2018 ICM criteria for PJI. This classification was based on both preoperative and postoperative data to align with the most widely adopted, expert consensus-based framework for PJI diagnosis in the United States. Three vignettes were excluded due to insufficient preoperative minor criteria, leaving 274 vignettes for inclusion. Of these, 47 met the 2018 ICM definition of PJI, 197 were aseptic, and 30 were classified as inconclusive (Table 1).

### 2.3. Physician Survey

The twelve physicians who participated in the survey study included four academic arthroplasty surgeons (AS), four community arthroplasty surgeons (CS), and four infectious disease specialists (ID). All participants were qualified to participate in this study based on their specialized training and experience in caring for arthroplasty patients [10]. Each physician reviewed the clinical vignettes and integrated the available laboratory and clinical information to diagnose PJI in a manner consistent with their routine clinical practice, representing the current SOC for PJI workup.

Physician performance was assessed in two stages. In Stage I, physicians assigned each vignette a diagnosis of PJI, aseptic, or undecided. This allowed the authors to quantify the proportion of initially uncertain diagnoses and to evaluate interobserver agreement. In Stage II, physicians re-evaluated only the cases they had previously labeled as undecided and were required to select either PJI or aseptic, ensuring that each vignette received a definitive diagnosis.

### 2.4. SynTuition Score

SynTuition provides an ML-based probability score of PJI using SF biomarkers as input. The SynTuition model was developed using a large multi-center dataset and a two-stage ML pipeline that combined linear and non-linear feature scaling, and unsupervised and supervised ML approaches. This enabled the authors to develop a model that was independent of predefined diagnostic criteria. A Gaussian Mixture Model (GMM) was used to find biologically meaningful clusters in a training cohort of 83,272 samples. These cluster labels were subsequently used to train a Logistic Regression model to provide a probability score. The SynTuition model was validated on an independent cohort of 20,818 samples, demonstrating high diagnostic accuracy without requiring culture results.

The SynTuition Score ranges from 0 to 100, with higher values indicating a greater probability of PJI. Thresholds established in the validation study classify scores ≥ 80 as a high probability of infection, scores < 20 as a low probability (aseptic), and intermediate values (20–80) as equivocal (undecided) [19].

A SynTuition Score was calculated for each of the 274 clinical vignettes included in this study. The SynTuition model requires 11 SF biomarkers: two specimen-integrity markers (absorbance at 280 nm wavelength [A280] and SF-RBC); three general inflammatory markers (SF-WBC, SF-PMN%, and synovial fluid CRP [SF-CRP]); one PJI-specific host-response marker (AD); and five direct microbial antigen detection markers (*Staphylococcus* targets [SPA and SPB], *Enterococcus* target [EF], *Candida* target [CP], and *Cutibacterium acnes* target [PAC]).

Four of the SF biomarker inputs, SF-PMN%, SF-WBC, AD, and SF-RBC, were available through the clinical vignettes. Of the 274 vignettes, 62% contained all four biomarkers, 97% contained at least three, and 99% contained at least two. Evaluation of the fully validated SynTuition model required all 11 biomarkers; therefore, missing biomarkers were imputed for vignettes with incomplete data. Imputation was performed using values derived from the original SynTuition training cohort [19]. Specifically, the mean of the scaled feature values was used, representing the central tendency of each biomarker used to train the SynTuition model. Because the original training cohort exhibited class imbalance, these mean values are biased by training samples labeled as Not Infected, thereby minimizing the risk that imputed values would artificially inflate scores for patients in this study. To validate this approach, mean-value imputation was evaluated in the original validation cohort, where it resulted in minimal impact on overall performance (~0.1%). When available, serum CRP values (present in 86.9% of cases) were substituted for SF-CRP, providing more informative input than assigning a fixed imputed value. Good correlation between serum CRP and SF-CRP has been observed in the literature [21], whilst the relatively low sensitivity of SF-CRP within the SynTuition model suggests that only large deviations from expected baseline values would meaningfully influence the model output [19]. Table 2 summarizes biomarker availability across all vignettes and the imputation values used for missing biomarkers.

For comparison with physician survey results, equivocal SynTuition Scores were reclassified as PJI when equal to or greater than 20 and as aseptic when less than 20, reflecting the threshold most likely used to guide cautious clinical decision-making when a definitive diagnosis is required. An important property of the SynTuition model is that it does not incorporate culture results and can be evaluated solely from SF biomarker results. Evaluation of the SynTuition Score was performed using Python 3.10 (Python Software Foundation, Beaverton, OR, USA) and scikit-learn 1.6.1.

### 2.5. Statistical Analysis

All statistical analyses and visualizations were performed in Python 3.10. Agreement between the clinical diagnosis and the physician groups was evaluated using overall percent agreement (OPA), positive percent agreement (PPA), and negative percent agreement (NPA) [22]. Gwet’s AC1 was used as an additional metric to account for chance agreement [23]. When comparing groups of physicians, pooled estimates were used. Confidence intervals for OPA, PPA, and NPA were calculated using the Wilson method [24]. Confidence intervals for Gwet’s AC1 were obtained via bootstrap resampling [25]. Decision curve analysis was used to illustrate the clinical utility of SynTuition when compared against the different physician groups [26].

### 2.6. Economic Impact

A decision-analytic model simulated a hypothetical cohort of 1000 suspected PJI cases. The costs of misdiagnosis were compared between the physician group, representing current SOC, and the SynTuition Score. For the simulation, the prevalence was set according to the study disease prevalence of 15.3% (Table 1), resulting in 153 PJI and 847 aseptic cases. The total number of simulated false-negative (FN) cases was calculated from the number of PJI cases and the PPA, where

(1) $$N_{FN}=153\times (1-PPA).$$

The number of simulated false-positive (FP) cases was calculated from the number of aseptic cases and the NPA, where

(2) $$N_{FP}=847\times (1-NPA)$$

The PPA and NPA for the physician group (SOC) were based on pooled results across all 12 participants in the physician survey study when compared against the clinical diagnosis.

The economic impact of a false-negative diagnosis was estimated using a scenario-based model reflecting two plausible downstream clinical pathways following an incorrect PJI negative result. To avoid reliance on variable or site-specific clinical distributions, the model assumes an equal probability of patients entering either pathway. In the first pathway, patients undergo additional nonoperative diagnostic evaluation, including joint aspiration, laboratory testing, and follow-up assessment. U.S. national price transparency data estimate the mean cost of a repeat diagnostic episode at approximately $1000, with a reported range of $500 to $2000 [27]. In the second pathway, delayed recognition of infection leads to operative management. As reported by Okafor et al., misdiagnosed patients commonly undergo an initial aseptic revision followed by a two-stage septic revision, resulting in a combined mean procedural cost of $123,750 [28]. Applying equal weighting to these pathways (0.50 × $1000 and 0.50 × $123,750) yields an expected false-negative cost of $62,375 per case.

The cost of a false-positive result was modeled as the cost of an unnecessary two-stage septic revision arthroplasty, estimated at $75,000 based on published U.S. revision cost analyses [28,29].

## 3. Results

### 3.1. Stage I Survey Results

Table 3 shows the distribution of PJI, aseptic, and undecided diagnoses based on Stage I of the physician survey. Of particular interest is the frequency of undecided diagnoses across different diagnostic methods and physician groups.

The 2018 ICM criteria classified 10.9% of cases as inconclusive, which aligns closely with the existing literature [30]. The rate of undecided diagnoses varied widely among physicians, ranging from 5.1% to 43.4%, reflecting the high level of uncertainty with the current standard of care. As previously reported, the level of uncertainty is influenced by physician experience. Academic surgeons, who are more familiar with PJI scoring systems, tend to have a lower indecision rate compared to community surgeons and ID physicians [10].

Due to the discriminative nature of the SynTuition algorithm, the proportion of uncertain results was significantly reduced to 0.4%. A similarly low rate of equivocal results has been previously reported in a large validation dataset [19].

### 3.2. Stage II Survey Results

Table 4 shows the distribution of PJI and aseptic diagnoses based on Stage II of the physician survey, in which all diagnostic methods and physicians were required to provide a definitive diagnosis. According to the clinical diagnosis, the prevalence of PJI across all 274 vignettes was 15.3%. Physicians consistently overestimated this prevalence, with an overall rate of 24.0%, suggesting that when required to make a definitive diagnosis, the current SOC tends to bias toward overdiagnosing PJI.

The agreement between each diagnostic method and the clinical diagnosis is provided in Table 5.

The SynTuition score demonstrated an OPA comparable to the academic surgeon group. Relative to the physicians using the current standard of care, SynTuition showed higher agreement, with an OPA of 96.0% and Gwet’s AC1 of 0.94 compared with 90.8% and 0.87, respectively.

While the NPA for SynTuition was higher than the current standard of care, at 96.6% compared to 89.4%, the PPA was lower (92.9% vs. 98.4%), indicating the presence of false-negative results relative to clinical diagnosis. These discrepant cases, with a select set of biomarkers and clinical information, are summarized in Table 6. Among these, FN-001 and FN-002 appear to represent true false-negative results, given their positive synovial fluid and tissue culture findings. SynTuition does not incorporate culture results; it is solely driven by the preoperative biomarkers. FN-001 has extremely low SF-WBCs, driving a low SynTuition Score, whilst FN-002 is predominantly driven by elevated CRP, known to be a relatively insensitive feature in the SynTuition model [19]. FN-003 is culture-negative and less concretely PJI with a high SF-RBC, raising the possibility of blood contamination in the SF, thus responsible for a low SynTuition Score.

Although these three cases lowered the SynTuition PPA, one is borderline or debatable, with a culture-negative result, and may even highlight the strengths of a machine learning approach that incorporates specimen integrity biomarkers [19]. Taken together, the performance of SynTuition remains credible given the imperfections of the clinical diagnosis being used.

### 3.3. 2018 ICM Inconclusive Cohort

Of particular interest is the performance of the diagnostic methods and physician groups in inconclusive or borderline cases, which are frequently associated with culture-negative results. Among the 30 cases classified as inconclusive by the 2018 ICM criteria (Table 1), 29 underwent synovial fluid culture and were all culture-negative. All 30 cases were aseptic according to the clinical diagnosis.

**Figure 2:**
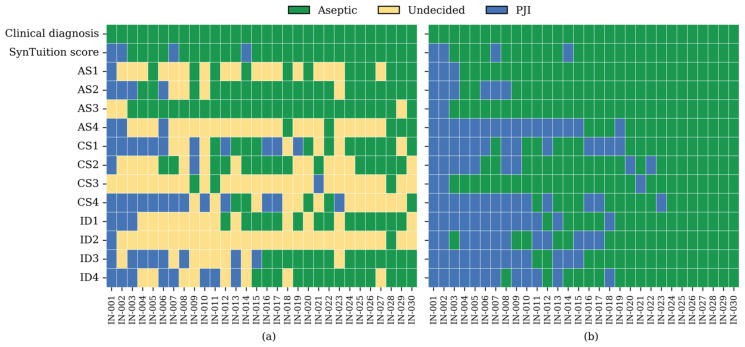
SynTuition Score and physician responses for the 2018 ICM inconclusive cohort. (**a**) Responses after stage I of the physician survey. (**b**) Responses after stage II of the physician survey when a definitive diagnosis was required. Abbreviations: IN, inconclusive; AS, academic surgeon; CS, community surgeon; ID, ID physician.

Figure 2 summarizes the diagnoses assigned by the SynTuition Score and each physician for the subset of cases classified as inconclusive by the 2018 ICM criteria. As shown in Figure 2a, a substantial proportion of physician responses were undecided during Stage I, with undecided rates of 38.3% among academic surgeons, 48.3% among community surgeons, and 47.5% among ID physicians, representing an overall SOC rate of 44.7% across all physicians. When restricted to responses requiring a definitive diagnosis, as shown in Figure 2b, the overall percent agreement (OPA) for SynTuition was 86.7%, compared with 77.5% for academic surgeons, 67.5% for community surgeons, 58.3% for ID physicians, and 67.8% for combined SOC. The high level of diagnostic uncertainty and lower OPA values observed across physician groups highlight the risk of both underdiagnosis and overdiagnosis within this challenging cohort.

### 3.4. Decision Curve Analysis

**Figure 3:**
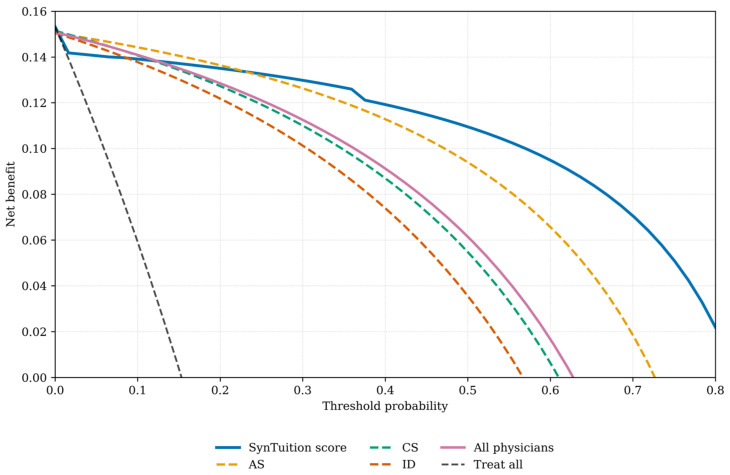
Decision curve analysis comparing the clinical utility of the SynTuition Score and physician groups. Abbreviations: AS, academic surgeon; CS, community surgeon; ID, ID physician.

Decision curve analysis is presented for each physician group and SynTuition (Figure 3). For each physician group, the mean net benefit across all threshold probabilities was calculated; for the all-physician group, the average net benefit across all 12 physicians was used. Interpretation of these curves requires selecting a threshold probability, with the diagnostic method showing the highest net benefit at that threshold, offering the greatest clinical value.

SynTuition demonstrated higher clinical value across a broad range of thresholds compared with the physician groups. For surgeons who prioritize treating every potential infection, even at the expense of overtreating some non-infected patients (threshold probability below 10%), the incremental benefit of SynTuition over current practice may be limited. However, many surgeons view a false-positive result as harmful and seek to avoid unnecessary treatment associated with overdiagnosis. Based on the decision curve analysis, surgeons with a threshold probability greater than or equal to 20% would benefit from incorporating the SynTuition Score into clinical decision-making.

**Figure 4:**
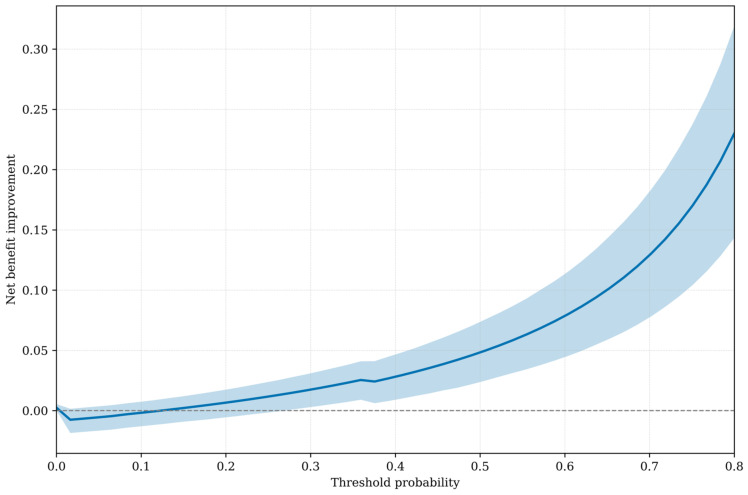
Net benefit improvement when comparing SynTuition and all physicians. The solid blue line is the mean net benefit improvement. The shaded blue region indicates the 95% bootstrap confidence interval. The dash line at zero denotes no difference in net benefit; values above this line indicate improved clinical utility of SynTuition relative to all physicians.

Figure 4 shows the net benefit SynTuition has over the SOC across a range of threshold probabilities. The solid line represents the mean net benefit improvement, while the shaded region denotes the 95% bootstrap confidence interval. The mean net benefit improvement increases with higher thresholds, and the 95% bootstrap confidence interval lies entirely above zero for threshold probabilities greater than approximately 30%.

To estimate a threshold probability reflecting economic value, we identified the point at which the expected cost of treatment equals the expected cost of no treatment, following the framework described by Vickers and Elkin [26]. This threshold corresponds to the ratio of the cost of a false-positive decision to the combined costs of false-positive and false-negative decisions. Using an estimated cost of $75,000 for a false positive and $62,375 for a false negative, the resulting threshold probability was 0.55 ($75,000 ÷ $137,375). At this threshold, SynTuition demonstrated a mean net benefit improvement of 0.06 (95% CI: 0.03 to 0.09) compared with current SOC. This improvement corresponds to approximately 4 to 11 fewer false positives per 100 patients, translating to a 4–11% reduction in unnecessary two-stage septic revision surgeries when SynTuition is applied at the economically optimal threshold.

### 3.5. Economic Impact Results

The economic implications of SynTuition were evaluated using a decision-analytic model simulating 1000 suspected PJI cases. Based on the SynTuition PPA and NPA (92.9% and 96.6%, Table 5) and an assumed cohort of 153 PJI and 847 aseptic cases, the model yielded 11 false negatives and 29 false positives. Using estimated costs of $62,375 per false-negative diagnosis and $75,000 per false-positive diagnosis, the total projected cost attributable to misdiagnosis was approximately $2.9 million.

In comparison, applying the PPA and NPA of the pooled physician group representing current SOC (98.4% and 89.4%, Table 5) resulted in 2 false negatives and 90 false positives. The disproportionately high number of false positives reflects a tendency among physicians to favor overdiagnosis when diagnostic uncertainty is present. Under these assumptions, the total cost of misdiagnosis is roughly $6.9 million. Comparing the two strategies, the reduction in misdiagnosis-related costs was $4.0 million, corresponding to an estimated net savings of $4000 per suspected PJI case when SynTuition is used as an adjunct to current SOC.

## 4. Discussion

In this study, we evaluated the diagnostic consistency and economic implications of SynTuition when compared against routine physician practice, which represents the current SOC. The results demonstrate that SynTuition provides a diagnostic performance comparable to, and in several respects exceeding, that of experienced physicians across orthopedic surgery and infectious disease specialties. Notably, the model substantially reduced diagnostic uncertainty and showed higher overall agreement with the clinical diagnosis than the pooled physician groups.

Across 274 clinical vignettes, SynTuition achieved an OPA of 96.0% and Gwet’s AC1 of 0.94, both of which were higher than those of the pooled all-physicians group (OPA: 90.8%, Gwet’s AC1: 0.87). Although the model’s PPA was lower than that of the pooled physicians, investigating discrepant cases identified that not all false-negative results represent clear misclassifications. Importantly, the SynTuition model does not incorporate culture results, and a score can be generated solely with synovial fluid biomarkers. This design does not preclude the use of culture data in clinical decision-making as and when it becomes available. While sequential decision-making was beyond the scope of this study, it is reasonable to suggest that a negative result by SynTuition could be reconsidered in the context of subsequent positive synovial fluid or tissue culture findings. Conversely, by operating independently of culture data and demonstrating a high NPA (96.6%), SynTuition supports early and reliable detection, with timely positive results facilitating the optimal selection of patients for less invasive interventions such as DAIR or DECRA (Debridement, Modular Exchange, Component Retention, Antibiotics).

A central challenge in PJI diagnosis is the management of borderline or inconclusive cases, which frequently arise in the context of culture-negative results. These situations can amplify the limitations of criteria-based systems and highlight variability in clinical decision-making. In this study, 10.9% of cases were classified as inconclusive by the 2018 ICM definition, which were all adjudicated as aseptic by a panel of three experts. Within this challenging cohort, SynTuition demonstrated no equivocal results, producing a definitive diagnosis in all cases, whilst achieving an OPA of 86.7%. In contrast, physicians utilizing SOC displayed high indecision rates (38–48%) and considerably lower agreement with the clinical diagnosis, with an OPA of 67.8% across all physicians. These findings suggest that the SynTuition Score provides meaningful support where ambiguity is greatest, helping to mitigate both under- and overdiagnosis. It should also be noted that although all uncertain cases in this study were adjudicated as aseptic, other investigations report higher proportions of borderline cases being reclassified as PJI (between 41% and 53%) [19,31].

Decision curve analysis further illustrated the clinical utility of SynTuition across a wide range of threshold probabilities. For physicians who place a high value on avoiding unnecessary revision procedures, particularly those who consider false-positive diagnoses harmful, the model provided a consistently greater net benefit than physician judgment alone. At an economically optimal threshold probability of 0.55, derived from current cost estimates for false-positive and false-negative diagnoses, SynTuition reduced unnecessary revision surgeries by an estimated 4–11 cases per 100 patients when compared with the pooled physician group. Although the decision curve analysis does not account for test-related harms, SynTuition carries no additional clinical risk beyond the current standard of care. Some physicians may nonetheless argue that its net benefit should be weighed against considerations of test cost and availability.

The economic analysis aligned with these observations. When applied to a simulated cohort of 1000 suspected PJI cases, SynTuition was projected to reduce misdiagnosis-related costs from roughly $6.9 million to $2.9 million, corresponding to estimated per-patient savings of $4000. Most of this reduction comes from the lower rate of false-positive diagnoses, reflecting a key advantage over physician tendencies toward overdiagnosis in ambiguous cases. These findings are based on a U.S. payer perspective, consistent with current SynTuition availability, and may not generalize to regions with differing healthcare cost structures or reimbursement models.

Despite the promising results, several limitations warrant consideration. First, although the vignettes were derived from real patient data and validated in prior work, they cannot fully replicate the richness of in-person clinical evaluation and nuanced contextual factors that influence diagnostic reasoning. However, the independent expert panel of adjudicators confirmed that sufficient information was available to render a conclusive diagnosis for each vignette. Second, some biomarkers required by the SynTuition model were missing from the vignette dataset and were imputed using values from the model’s training cohort. However, previous clinical validation studies demonstrated that the three most influential features for the model are AD, SF-WBC, and SF-PMN%, and these were present in over 96% of the dataset [19]. Third, physicians had access to information such as synovial fluid culture results and clinical context not available to SynTuition, while the model used variables not reviewed by physicians, such as AD and SF-RBC, that are not widely available under current standard diagnostic protocols. Fourth, the adjudicated 2013 MSIS-based classification remains an imperfect ground truth, and borderline classifications remain debatable. Nonetheless, in the absence of a gold standard, this represents the best available clinical diagnosis. Finally, this analysis relied on pooled statistics with wide variance and economic estimates from the published literature. Real-world costs will vary across institutions depending on clinical expertise, available resources, and established diagnostic pathways.

Future work may aim to evaluate SynTuition prospectively in clinical settings, incorporating sequential decision-making based on when biomarker results and other clinical information become available. The influence of SynTuition on downstream decision-making, including selection of DAIR/DECRA versus revision strategies, antibiotic stewardship, and outcomes, should also be examined.

## 5. Conclusions

This study demonstrates that SynTuition reduces diagnostic uncertainty, improves agreement with the clinical diagnosis, and offers meaningful clinical and economic advantages over the current standard-of-care diagnosis in suspected PJI. These findings support the role of machine learning-based diagnostic tools as valuable additions to clinical decision-making, particularly in cases where ambiguity poses a substantial risk to patient outcomes.

## Figures and Tables

**Table 1:** Distribution of clinical vignettes and PJI classification according to the clinical diagnosis and 2018 ICM criteria.

Diagnosis	Clinical Diagnosis	2018 ICM
PJI	42 (15.3%)	47 (17.2%)
Aseptic	232 (84.7%)	197 (71.9%)
Inconclusive	none	30 (10.9%)

**Table 2:** Availability of SynTuition-required biomarkers across 274 vignettes and imputed values used in place of missing SynTuition biomarkers. Serum CRP was used in place of SF-CRP. Abbreviations: AD, alpha-defensin; SF-WBC, synovial fluid white blood cell count; SF-PMN%, synovial fluid polymorphonuclear percentage; SF-RBC, synovial fluid red blood cell count; CRP, C-reactive protein; A280, absorbance at 280 nm wavelength; CP, *Candida* target; EF, *Enterococcus* target; SPA and SPB, *Staphylococcus* targets; PAC, *Cutibacterium acnes* target.

Biomarker	Availability, *n* (%)	Imputed Value
AD	273 (99.6%)	0.196
SF-WBC	268 (97.8%)	843
SF-PMN%	265 (96.7%)	48
SF-RBC	174 (63.5%)	18,895
CRP	238 (86.9%)	1.7
A280	0	0.643
CP	0	0.48
EF	0	0.5
SPA	0	0.73
SPB	0	0.46
PAC	0	0.1

**Table 3:** Distribution of PJI, aseptic, and undecided diagnoses across diagnostic methods and physicians. Abbreviations: PJI, periprosthetic joint infection; ICM, international consensus meeting; AS, academic surgeons; CS, community surgeons; ID, infectious disease specialists.

Diagnostic Method/Physician	PJI, *n* (%)	Aseptic, *n* (%)	Undecided, *n* (%)
Clinical diagnosis	42 (15.3%)	232 (84.7%)	none
2018 ICM	47 (17.2%)	197 (71.9%)	30 (10.9%)
SynTuition Score	46 (16.8%)	227 (82.8%)	1 (0.4%)
All physicians	597 (18.2%)	1937 (58.9%)	754 (22.9%)
Academic surgeons	172 (15.7%)	751 (68.6%)	173 (15.8%)
AS1	41 (15.0%)	191 (69.7%)	42 (15.3%)
AS2	49 (17.9%)	211 (77.0%)	14 (5.1%)
AS3	35 (12.8%)	223 (81.4%)	16 (5.8%)
AS4	47 (17.2%)	126 (46.0%)	101 (36.9%)
Community surgeons	220 (20.1%)	563 (51.4%)	313 (28.6%)
CS1	67 (24.5%)	150 (54.7%)	57 (20.8%)
CS2	44 (16.1%)	170 (62.0%)	60 (21.9%)
CS3	42 (15.3%)	141 (51.5%)	91 (33.2%)
CS4	67 (24.5%)	102 (37.2%)	105 (38.3%)
ID physicians	205 (18.7%)	623 (56.9%)	268 (24.5%)
ID1	47 (17.2%)	176 (64.2%)	51 (18.6%)
ID2	40 (14.6%)	115 (42.0%)	119 (43.4%)
ID3	62 (22.6%)	184 (67.2%)	28 (10.2%)
ID4	56 (20.4%)	148 (54.0%)	70 (25.5%)

**Table 4:** Distribution of PJI and aseptic diagnoses across diagnostic methods and physicians. Abbreviations: PJI, periprosthetic joint infection; AS, academic surgeons; CS, community surgeons; ID, infectious disease specialists.

Diagnostic Method/ Physician	PJI, *n* (%)	Aseptic, *n* (%)
Clinical diagnosis	42 (15.3%)	232 (84.7%)
SynTuition Score	47 (17.2%)	227 (82.8%)
All physicians	790 (24.0%)	2498 (76.0%)
Academic surgeons	227 (20.7%)	869 (79.3%)
AS1	49 (17.9%)	225 (82.1%)
AS2	54 (19.7%)	220 (80.3%)
AS3	45 (16.4%)	229 (83.6%)
AS4	79 (28.8%)	195 (71.2%)
Community surgeons	272 (24.8%)	824 (75.2%)
CS1	81 (29.6%)	193 (70.4%)
CS2	61 (22.3%)	213 (77.7%)
CS3	55 (20.1%)	219 (79.9%)
CS4	75 (27.4%)	199 (72.6%)
ID physicians	291 (26.6%)	805 (73.4%)
ID1	74 (27.0%)	200 (73.0%)
ID2	69 (25.2%)	205 (74.8%)
ID3	74 (27.0%)	200 (73.0%)
ID4	74 (27.0%)	200 (73.0%)

**Table 5:** Agreement between diagnostic methods and the clinical diagnosis. Overall percent agreement (OPA), positive percent agreement (PPA), negative percent agreement (NPA). Values for each physician group and all physicians group are based on pooled results. Abbreviations: AS, academic surgeons; CS, community surgeons; ID, infectious disease specialists; CI, confidence interval.

Diagnostic Method/Physician	OPA (95% CI)	PPA (95% CI)	NPA (95% CI)	Gwet’s AC1 (95% CI)
SynTuition Score	96.0% (93.0–97.7%)	92.9% (81.0–97.5%)	96.6% (93.3–98.2%)	0.94 (0.91–0.98)
All physicians	90.8% (89.8–91.8%)	98.4% (96.9–99.2%)	89.4% (88.2–90.5%)	0.87 (0.85–0.88)
Academic surgeons	94.1% (92.5–95.3%)	98.2% (94.9–99.4%)	93.3% (91.5–94.8%)	0.92 (0.89–0.94)
AS1	97.4% (94.8–98.8%)	100.0% (91.6–100.0%)	97.0% (93.9–98.5%)	0.96 (0.94–0.99)
AS2	95.6% (92.5–97.5%)	100.0% (91.6–100.0%)	94.8% (91.2–97.0%)	0.94 (0.90–0.97)
AS3	97.4% (94.8–98.8%)	95.2% (84.2–98.7%)	97.8% (95.1–99.1%)	0.97 (0.94–0.99)
AS4	85.8% (81.1–89.4%)	97.6% (87.7–99.6%)	83.6% (78.3–87.8%)	0.78 (0.71–0.85)
Community surgeons	90.1% (88.2–91.8%)	98.8% (95.8–99.7%)	88.6% (86.4–90.5%)	0.85 (0.83–0.88)
CS1	85.8% (81.1–89.4%)	100.0% (91.6–100.0%)	83.2% (77.8–87.5%)	0.78 (0.71–0.85)
CS2	93.1% (89.4–95.5%)	100.0% (91.6–100.0%)	91.8% (87.6–94.7%)	0.90 (0.85–0.94)
CS3	93.8% (90.3–96.1%)	95.2% (84.2–98.7%)	93.5% (89.6–96.0%)	0.91 (0.87–0.95)
CS4	88.0% (83.6–91.3%)	100.0% (91.6–100.0%)	85.8% (80.7–89.7%)	0.82 (0.75–0.88)
ID physicians	88.2% (86.2–90.0%)	98.2% (94.9–99.4%)	86.4% (84.1–88.5%)	0.82 (0.79–0.85)
ID1	87.6% (83.2–91.0%)	97.6% (87.7–99.6%)	85.8% (80.7–89.7%)	0.81 (0.75–0.87)
ID2	89.4% (85.2–92.5%)	97.6% (87.7–99.6%)	87.9% (83.1–91.5%)	0.84 (0.78–0.9)
ID3	87.6% (83.2–91.0%)	97.6% (87.7–99.6%)	85.8% (80.7–89.7%)	0.81 (0.74–0.87)
ID4	88.3% (84.0–91.6%)	100.0% (91.6–100.0%)	86.2% (81.2–90.1%)	0.82 (0.76–0.88)

**Table 6:** Biomarker and clinical information associated with the three false-negative results when comparing the SynTuition score against the clinical diagnosis. Abbreviations: FN, false-negative; CRP, C-reactive protein; SF-WBC, synovial fluid white blood cell count; SF-RBC, synovial fluid red blood cell count; SF-PMN%, synovial fluid polymorphonuclear percentage; AD, alpha-defensin.

	FN-001	FN-002	FN-003
CRP	15	200.4	1
SF-WBC	40	200	5354
SF-RBC	imputed	imputed	786,000
SF-PMN%	72	42	93
AD	1.599	0.34	0.156
Culture	positive	positive	negative
Joint	hip	knee	hip

## Data Availability

The data supporting the findings of this study are available from the corresponding author upon reasonable request. Because the dataset includes patient test data, public sharing could pose a risk of re-identification and violate HIPAA privacy regulations. Requests will be reviewed to ensure compliance with applicable privacy laws and institutional policies, and data will only be shared for legitimate research purposes.

## Abbreviations

The following abbreviations are used in this manuscript:

PJI	Periprosthetic joint infection
DAIR	Debridement, antimicrobial therapy, and implant retention
DECRA	Debridement, Modular Exchange, Component Retention, Antibiotics
SOC	Standard of care
PPA	Positive percent agreement
NPA	Negative percent agreement
OPA	Overall percent agreement
AD	Alpha defensin
SF-WBC	Synovial fluid white blood cell
SF-PMN%	Synovial fluid polymorphonuclear percentage
SF-RBC	Synovial fluid red blood cell
SF-CRP	Synovial fluid C-reactive protein
A280	Spectrophotometric absorbance at 280 nm wavelength
CP	*Candida* microbial antigen
EF	*Enterococcus* microbial antigen
SPA	*Staphylococcus* microbial antigen A
SPB	*Staphylococcus* microbial antigen B
PAC	*Cutibacterium acnes* microbial antigen
